# Effects of Juglone on Neutrophil Degranulation and Myeloperoxidase Activity Related to Equine Laminitis

**DOI:** 10.3389/fvets.2021.677675

**Published:** 2021-07-16

**Authors:** Ange Mouithys-Mickalad, Nazaré Storms, Thierry Franck, Justine Ceusters, Geoffroy de la Rebière de Pouyade, Ginette Deby-Dupont, Didier Serteyn

**Affiliations:** ^1^Centre for Oxygen R&D, Institute of Chemistry, B6a, University of Liège, Liège, Belgium; ^2^Department of Clinical Sciences, Equine Surgery, University of Liège, Liège, Belgium

**Keywords:** neutrophil degranulation, myeloperoxidase, juglone, reactive oxygen species, electron paramagnetic resonance spectroscopy

## Abstract

Experimental laminitis, characterized by a failure of the dermal–epidermal interface of the foot, can be induced in horses by the oral administration of a black walnut extract (BWE). In the early phase of this severe and painful disease, an activation of neutrophil occurs, with the release of myeloperoxidase (MPO), a pro-oxidant enzyme of neutrophils, in plasma, skin, and laminar tissue. Juglone, a naphthoquinone derivative endowed with redox properties, is found in walnuts and has been incriminated in this neutrophil activation. We report for the first time the inhibitory activity of juglone on the degranulation of neutrophils induced by cytochalasin B and formyl-methionyl-leucyl-phenylalanine as monitored by the MPO release (>90% inhibition for 25 and 50 μM). Moreover, it also acts on the peroxidase activity of MPO by interacting with the intermediate “π cation radical,” as evidenced by the classical and specific immunological extraction followed by enzymatic detection (SIEFED) assays. These results are confirmed by a docking study showing the perfect positioning of juglone in the MPO enzyme active site and its interaction with one of the amino acids (Arg-239) of MPO apoprotein. By chemiluminescence and electron paramagnetic resonance techniques, we demonstrated that juglone inhibited reactive oxygen species (ROS) and superoxide anion free radical produced from phorbol myristate acetate (PMA)-activated polymorphonuclear neutrophils (PMNs). These results indicate that juglone is not the trigger for equine laminitis, at least if we focus on the modulation of neutrophil activation.

## Introduction

Laminitis is a common and very severe disease of the foot of horses, characterized by a failure of the dermal–epidermal interface that induces dramatic pain and, in severe cases, requires euthanasia (1, 2). Laminitis results from a combination of factors most often generated during an excessive and systemic inflammatory reaction, such as decreased blood flow (ischemia) and inflammation in the foot involving endothelial/vascular dysfunctions, degradation of the extracellular matrix, and metabolic abnormalities in keratinocytes (3–5). The activation of neutrophils (polymorphonuclear neutrophils, PMNs) plays an important role in the initial stages of laminitis (6, 7). Two models are usually used in research to induce experimental laminitis: an oral administration of blackwalnut extract (BWE) simulating sepsis or a hyperinsulinemia model to mimic endocrinopathic laminitis (8–12). Our group previously demonstrated neutrophil activation in a BWE model of laminitis: myeloperoxidase (MPO), a pro-oxidant enzyme of neutrophils, and neutrophil elastase were present in plasma, skin, and laminar tissue in correlation with the emigration of white blood cells from the vasculature, supporting a role for neutrophil activation and systemic inflammation in the early phase of the disease (13, 14). More recently, similar pictures were obtained with the hyperinsulinemia model (unpublished work).

Several teams have considered that juglone, present in some BWE, could be the trigger capable of inducing laminitis, but other groups did not find this compound in active BWE (8, 12, 15). In order to resolve this controversy, this work was designed to study the effects of juglone on the activation of neutrophils involved in the pathophysiology of equine laminitis regardless of the mechanism responsible for the onset of the disease.

Juglone (5-hydroxy-1,4-naphthoquinone), a phenolic compound found in walnuts, has been extensively studied not only for its redox properties but also for its antimicrobial properties and its implication in anticancer activity through the signaling pathway and reactive oxygen species (ROS) production (16–22). On the basis of *in vitro* studies, juglone has been described to inhibit the respiration rate on a cellular model (keratinocytes) in conjunction with the formation of semiquinone intermediate radicals (23). It is well-known that the generation of ROS and the modulation of redox signaling are properties of quinones (21, 24). The cytotoxic properties of naphthoquinones, like juglone, involve the induction of oxidative stress with redox cycling, cell membrane damage, apoptosis, and necrotic cell death (25, 26). However, other authors like Dehorty and his group reported that redox cycling did not play a role in the mechanism of the toxicity of naphthoquinone derivatives, including juglone (27), raising the question of its action depending on the type of cells and pathological situation.

An increasing body of interest has been focused on the mechanism of action of neutrophil degranulation, but little is known regarding the effect of juglone on the activity of MPO, a neutrophil oxidant enzyme also considered as a marker of inflammation (28, 29).

This work investigated the direct effect of juglone on the equine neutrophils activated by the cytochalasin B (CB)/formyl-methionyl-leucyl-phenylalanine (fMLP) system or phorbol 12-myristate 13-acetate (PMA) in order to verify whether or not juglone can trigger ROS production and neutrophil degranulation and act on the release and activity of MPO. We also compared the effects of juglone on equine MPO activity as well as on human MPO activity before performing docking assays on human MPO.

## Materials and Methods

### Reagents

All the reagents were of analytical grade. Dimethyl sulfoxide (DMSO), ethanol, CaCl_2_, KCl, NaCl, hydrogen peroxide (H_2_O_2_) 30%, and Tween-20 were all supplied by Merck (VWRI, Leuven, Belgium). Sodium nitrite (NaNO_2_), bovine serum albumin (BSA), PMA, gallic acid, CB, and fMLP were all purchased from Sigma-Aldrich (Bornem, Belgium). Percoll was from GE Healthcare (VWR, Leuven, Belgium). 8-Amino-5-chloro-7-phenylpyrido[3,4-d]pyridazine-1,4(2H,3H)dione (L-012) was from Wako Chemicals (Neuss, Germany) and Trypan blue was from ICN Biomedicals, Inc. (Aurora, OH, USA). MTS Cell Titer 96^R^ was purchased from Promega REF G5430 (Madison, WI, USA), and human MPO (200 U/mg protein) was obtained from Calbiochem Millipore (Billerica, MA, USA). Equine MPO was purified according to the procedure previously reported (30). Juglone was obtained from Cayman Chemical Company (Ann Arbor, MI, USA). Amplex Red was from Molecular Probes Inc. (Leiden, Netherlands). 5,5′-Dimethyl-pyrroline-*N*-oxide (DMPO) was obtained from Enzo Life Sciences (Brussels, Belgium). All aqueous solutions were prepared with water previously purified in a Milli-Q water system (Millipore, Bedford, MA, USA).

### Preparation of Juglone Solutions

The stock solution of juglone was prepared at a concentration of 10 mM by dissolving (1.74 mg/ml) in DMSO. Appropriate dilutions were then performed to obtain 1, 0.1, 0.01 mM, etc., by using DMSO as a solvent and not as a buffer in order to avoid any precipitate at the highest juglone concentrations. To obtain the final concentrations in the reaction vials, each dilution was diluted 100 times with buffer solution. DMSO was thus at 1% in all the samples at the final dilution.

### Equine Neutrophil Isolation and Juglone Toxicity Assay on Neutrophils

Equine neutrophils were isolated from whole blood using EDTA disodium salt (1.6 mg/ml) as anticoagulant. The blood was drawn from the jugular vein of healthy horses bred and fed under identical conditions and without medical treatment. All the experiments were realized with approval from the ethics committee of the Faculty of Veterinary Medicine of the University of Liege (agreement no. 1474). Briefly, the neutrophils were isolated at room temperature (18–22°C) by centrifugation (400 × *g*, 45 min at 20°C) on a discontinuous Percoll density gradient according to the method previously described (31). The cells were gently collected and washed with two volumes of physiological saline solution. After removal of the supernatant, the cell pellets were resuspended in 2 ml phosphate-buffered saline (PBS) and counted for further use.

A cell viability test was performed by using the Trypan blue exclusion by viable cells, as described by Strober (32). The samples were prepared as follows: PMNs were incubated for 30 min with juglone at different concentrations. Afterwards, the cellular suspension was centrifuged and the supernatant eliminated. The cell pellets were resuspended in 450 μl PBS, to which was added 50 μl of Trypan blue. Ten microliters of the cell suspension was transferred into the Bürker chamber for colored dead cell counting under light microscopy.

### Neutrophil ROS Production

#### Luminescence Investigation

The ROS produced by PMA-activated neutrophils were measured by L012-enhanced chemiluminescence (CL) under adaptation of the method previously described by Benbarek et al. (31) and Derochette et al. (33). Neutrophil suspensions were distributed in the wells (10^6^ neutrophils/well, 143 μl PBS) of a 96-well microtiter plate (White Combiplate 8; Fisher Scientific, Merelbeke, Belgium) and incubated for 10 min at 37°C with 2 μl of juglone to reach final concentrations of 0.001, 0.01, 0.1, and 0.5 μM. After incubation, 25 μl CaCl_2_ (10 mM) and 20 μl L-012 (10^−4^ M) were added into the wells (final volume, 200 μl). Then, the suspensions were activated with 10 μl PMA (16 μM) just before CL measurement (33). The CL response of the neutrophils was monitored for 30 min at 37°C with a Fluoroskan Ascent spectrophotometer (Fisher Scientific, Merelbeke, Belgium) and expressed as the integral value of the total CL emission. The control was performed with neutrophils activated with PMA [positive control (Ctrl)] in the presence of PBS instead of juglone. Another control was performed with PMA-activated neutrophils in the presence of the vehicle solution of juglone (1% DMSO, final concentration) and was taken as 100% CL response. The negative control was done with unstimulated neutrophils (non-activated, NA) with the CL probe alone.

#### EPR Spin Trapping Investigation

In parallel to the CL assay, the effect of juglone on ROS production by PMA-activated neutrophils was evidenced by using electron paramagnetic resonance (EPR) spectroscopy in combination with the spin trapping technique (DMPO was used as the spin trap agent). The EPR assay was performed according to the protocol previously described (34). Neutrophil suspensions were distributed in Eppendorf tubes (4 × 10^6^ neutrophils/ml, 143 μl PBS) in the presence of 10 μl of DMPO (50 mM) and 5 μl of CaCl_2_ (10 mM). The reaction was triggered upon the addition of 10 μl PMA (5 × 10^−7^ M). The solutions of juglone were tested at final concentrations of 0.5, 1, 2.5, and 10 μM and compared to the complete system without juglone or with 1% DMSO used as a vehicle control. The sample was then transferred in the capillary, which was put into the EPR quartz tube and placed into the cavity for measurement using an EMX-micro EPR (Bruker, Rheinstetten, Germany). The following settings were used for the analysis: microwave frequency, 9.78 GHz; microwave power, 18.9 mW; modulation amplitude, 1.0 G; modulation frequency, 100 kHz; receiver gain, 2 × 10^4^; conversion time, 40 ms; time constant, 81.92 ms; magnetic field centered at 3,480 G; and number of scans, 4.

### Neutrophil Degranulation Activity

The active MPO fraction released by PMNs was measured by the specific immunological extraction followed by enzymatic detection (SIEFED) method (35). Briefly, neutrophils (10^6^ cell/ml) in 20 mM PBS (pH 7.4) were incubated for 10 min at 37°C with 5 μl CB (1 mg/ml) in the presence or absence of increasing concentrations of juglone (0.1, 0.5, 1, 2.5, 5, 10, and 50 μM) and 1% DMSO used as a vehicle control. Then, the cell suspensions were stimulated with 1 × 10^−6^ M fMLP (36). A negative control assay was performed with neutrophils without any addition of juglone and stimulating agents. All the samples were incubated at 37°C for 30 min and then centrifuged at 450 × *g* for 10 min at 37°C. The supernatant was collected and transferred into a 5-ml tube and stored at −20°C until further measurement of the active MPO.

### Myeloperoxidase Activity

#### Purification of Equine MPO

Pure equine MPO was obtained as previously described by Franck et al. (30). Briefly, packed neutrophils were homogenized in acetate buffer (pH 4.7) added with 1% detergent. After the dialysis of the supernatant, MPO was purified by two successive chromatographic steps: ion exchange on Sepharose gel (acetate buffer, pH 4.7, NaCl gradient) and gel filtration (same acetate buffer). After the final dialysis, MPO was >98% pure (as established by electrophoresis with enzymatic detection on electrophoretic bands). The enzyme-specific activity determined by the *ortho*-dianisidine test at pH 5.5 was 70.4 U/mg protein.

#### Measurement of MPO Activity

Measurement of the peroxidase activity of MPO was performed with a classical enzymatic assay and the SIEFED assay as described by Nyssen et al. (37). The MPO solution was prepared with purified equine or human MPO in the dilution buffer (20 mM PBS, pH 7.4, with 5 g/L BSA and 0.1% Tween-20). The solutions of juglone, at final concentrations ranging from 0.1 to 50 μM, were incubated for 10 min with equine or human MPO at a final concentration of 5 mU/ml before further use. The revelation of MPO activity was performed by monitoring the enzyme-catalyzed oxidation of Amplex Red in the presence of H_2_O_2_ (10 μM) and nitrite (4.5 mM) in phosphate buffer, pH 7.4. Similar assays were performed in the same conditions with gallic acid instead of juglone. Gallic acid was used at final concentrations ranging from 0.1 to 0.5 μM, dissolved in Milli-Q-distilled H_2_O; this molecule was chosen as a positive control for its known inhibitory properties on MPO activity (38).

##### Classical Assay of MPO Activity

After incubation, the mixtures containing 100 μl of juglone/vehicle and MPO were loaded into the wells of microtiter plates (transparent) and the peroxidase activity measured by adding 10 μl sodium nitrite solution (4.5 mM, final concentration) and 100 μl of the reaction solution containing 10 μM H_2_O_2_ and 40 μM Amplex® Red in phosphate buffer (50 mM) at pH 7.5. The oxidation of Amplex® Red into the fluorescent adduct resorufin (λ_excitation_ = 544 nm, λ_emission_ = 590 nm) was monitored for 30 min at 37°C with a fluorescent plate reader (Fluoroskan Ascent, Fisher Scientific). A control assay set as relative percentage value of MPO activity was performed with purified MPO in the presence of PBS instead of the increasing concentrations of juglone and 1% DMSO used as the solvent of juglone. To eliminate the possibility of artifact reactions, which might arise from the MPO activity or its natural substrate (H_2_O_2_), direct reaction of juglone with H_2_O_2_ was performed in phosphate buffer (PBS) without the addition of equine or human MPO.

##### SIEFED Assay of MPO Activity

Samples with MPO and various concentrations of juglone were prepared and incubated as for the classical assay. One hundred microliters of each mixture (MPO alone or MPO + juglone/DMSO) was then loaded into the wells of a SIEFED microtiter plate coated with rabbit polyclonal antibodies (3 μg/ml) against equine MPO or against human MPO and incubated for 2 h at 37°C in darkness. After washing up the wells, the activity of the enzyme captured by the antibodies was measured by adding 10 μl sodium nitrite solution (4.5 mM, final concentration) and 100 μl of a reaction solution containing 10 μM H_2_O_2_ and 40 μM Amplex® Red in phosphate buffer (50 mM) at pH 7.5. The oxidation of Amplex® Red into the fluorescent adduct resorufin (λ_excitation_ = 544 nm, λ_emission_ = 590 nm) was monitored for 30 min at 37°C with a fluorescent plate reader (Fluoroskan Ascent, Fisher Scientific). As for the MPO direct assay, a control assay set as relative value of MPO activity was performed with purified MPO in the presence of PBS instead of the samples of juglone dissolved in DMSO. In this SIEFED assay, MPO was bond by the antibodies into the wells and juglone was discarded by the washing step before starting the measurement of the enzymatic activity of the enzyme.

### Docking of MPO–Juglone

Docking simulations were realized to determine the capacity of juglone to enter into the active site of the human enzyme MPO, based on the inhibitory effect on MPO activity previously analyzed by SIEFED, and to investigate its binding mode with amino acids near the heme cavity. This assay was performed on the human MPO as its crystallographic (X-ray) structure is well-known in contrast to that of equine MPO (39). The potential inhibitory effect of juglone was docked in the heme pocket using the GOLD program as described by Nyssen et al. (37). Five runs have been performed with the aim to determine the most frequent solutions and ensure their reproducibility.

### Statistical Analysis

Data are given in relative values (in percent) in reference to control groups (DMSO or distilled H_2_O) defined as 100%. All data are expressed as the mean ± standard error of the mean (SEM) of at least two independent experiments made with different cell batches: in each independent experiment, the assays were performed in duplicate. For the MPO activity, the assays were performed in quadruplicate. Statistical analysis was performed with the corresponding solvent vehicle control group as the reference. One-way ANOVA with Dunnett's multiple comparison test was performed. A *p* < 0.05 was considered as significant.

## Results

### Neutrophil Toxicity

The Trypan blue test showed that the viability of the cells was not significantly affected after 40 min, which corresponds to the total incubation time used when neutrophils were treated with various concentrations of juglone during the stimulating assays (CL and degranulation). Only a weak cellular toxicity is observed upon exposure to the highest concentrations of juglone: 10–20% loss of viable cells at the tested concentrations of juglone (from 0.001 to 50 μM).

### Neutrophil ROS Production

#### Luminescence Investigation

**Figure 1:**
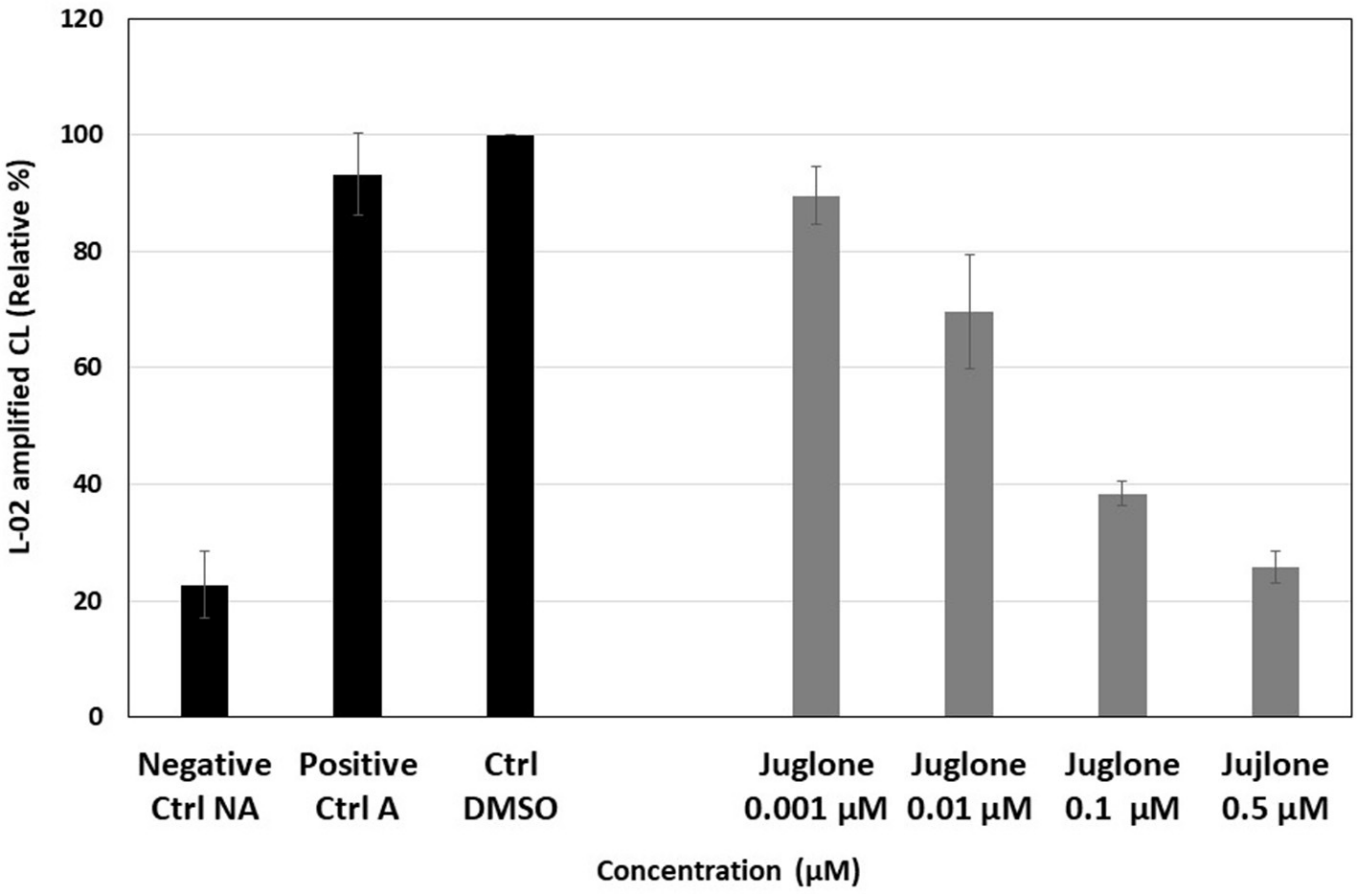
Effect of increasing concentrations (0.001–0.5 μM) of juglone on the reactive oxygen species (ROS) produced by phorbol myristate acetate (PMA)-activated neutrophils (1 million/well in PBS in the presence of 10 mM calcium and the chemiluminescent probe, 10^−4^ M L-012). The results are the mean ± SD of triplicate experiments (*n* = 6). *Negative Ctrl (NA)*, non-activated cells (no PMA); *positive Ctrl (A)*, PMA-activated cells (+*PMA*); *Ctrl DMSO*, control of activated cells with 1% DMSO taken as 100% of ROS production.

The CL response resulting from ROS production by PMA-stimulated PMNs was inhibited by juglone in a dose-dependent manner, and this inhibition reached 78% for the highest concentration of 0.5 μM. In the absence of PMA (NA neutrophils, negative Ctrl NA), only a weak light emission was observed compared to activated neutrophils (A, positive Ctrl A) with PMA. The control test with 1% DMSO, used as the solvent of juglone, did not induce a significant change compared to the positive control (Figure 1). In contrast, upon the exposure of juglone with PMNs without PMA, in the presence of the CL probe (L-012), no ROS production was observed (data not shown).

#### EPR Spin Trapping Investigation

**Figure 2:**
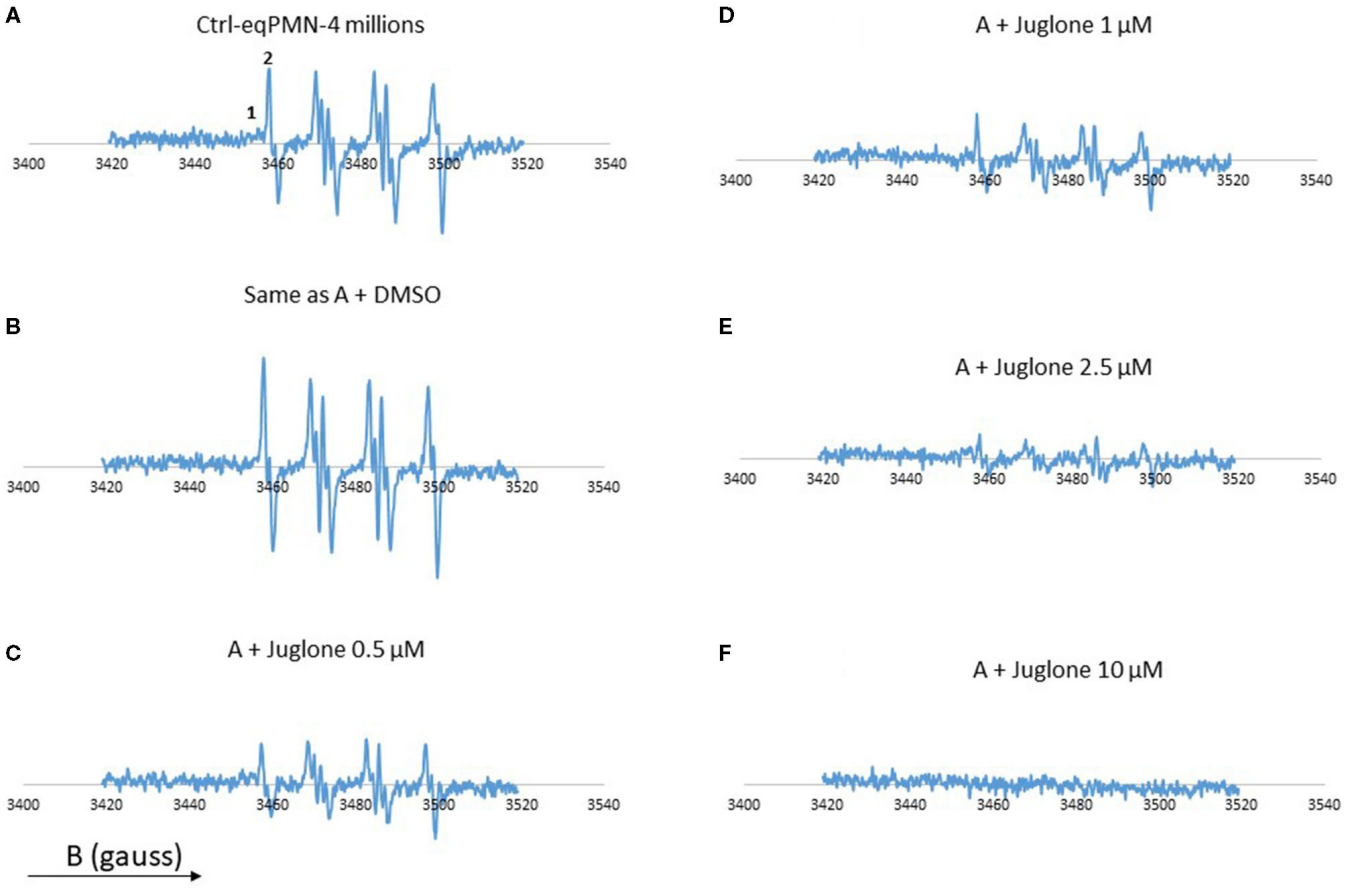
Electron paramagnetic resonance (EPR) spectra from neutrophils (4 × 10^6^ cells/ml) stimulated with phorbol myristate acetate (PMA, 5 × 10^−7^ M) in the presence of the spin trap 5,5′-dimethyl-pyrroline-*N*-oxide (DMPO, 100 mM). Scan **(A)** complete system which generates oxygen free radicals. Scan **(B)** same as **(A)**, but with 1% DMSO. Scan **(C)** same as **(A)**, but with 0.5 μM juglone. Scan **(D)** same as **(A)** + juglone 1 μM. Scan **(E)** same as **(A)** + juglone 2.5 μM. Scan **(F)** same as **(A)** + juglone 10 μM. Hyperfine splitting for scan **(A)** a*H* = 14·3 G and a*N* = 14·87 G. The results represent the mean of two experiments with two different cell batches. (1) DMPO–OH adduct. (2) DMPO–OOH adduct. The total number of scans is 4.

In the absence of juglone, a strong EPR signal was observed, which was a little bit increased in the presence of 1% DMSO (Figure 2). In contrast, when PMA-activated neutrophils were pre-incubated with increasing concentrations of juglone (from 0.5 to 10 μM), a decrease of the EPR signal was observed in a dose-dependent manner, with total disappearance at the highest concentration of 10 μM (Figure 2). The EPR signal resulting from the activation of PMNs is a mixture of two EPR spectra: a very weak signal attributed to the DMPO–OH adduct (1 in Figure 2, scan A) and the high signal of the DMPO–OOH adduct (2 in Figure 2, scan A). As expected, no EPR signal was observed with the non-activated neutrophils (not shown).

### Neutrophil Degranulation Activity

**Figure 3:**
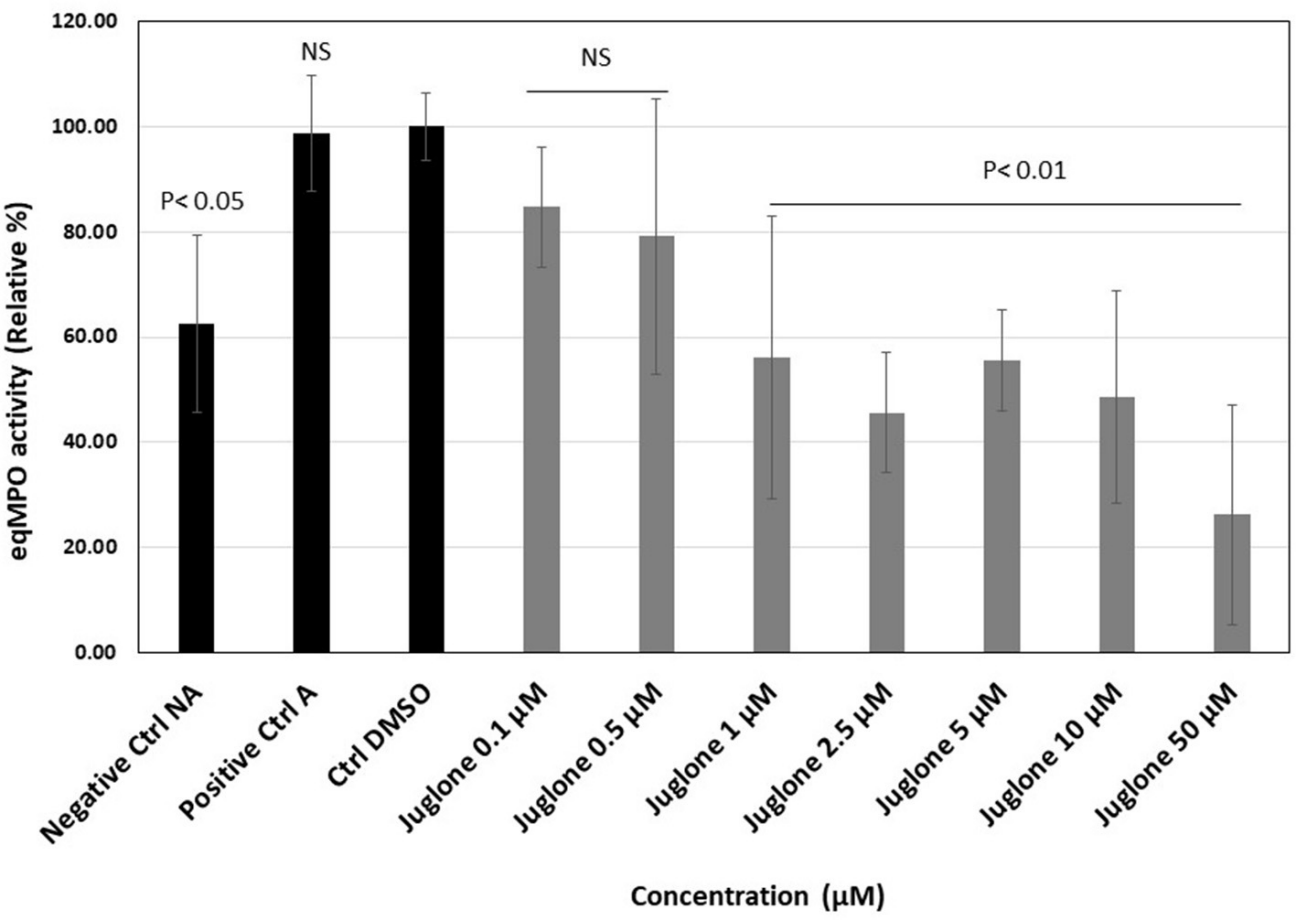
Effect of juglone on the active myeloperoxidase (MPO) release during the degranulation of neutrophils in the absence or presence of increasing concentrations of juglone (from 0.1 to 50 μM). *Positive control (A)*, activated neutrophils alone; *Ctrl DMSO*, control with 1% dimethyl sulfoxide (DMSO). The results are presented as the mean value ± SD of independent experimental conditions (*n* = 6). The mean value obtained for the condition Ctrl DMSO was taken as 100%. *p* < 0.05 and *p* < 0.01 were considered as significant.

The activation of neutrophils (10^6^ cells/ml) with the mixture of CB and fMLP induced a strong release of active MPO measured in the cell supernatant. Figure 3 presents, for each experimental condition, the individual values obtained for six batches of neutrophils isolated from different horses. Upon exposure to increasing concentrations of juglone (from 0.1 to 50 μM), a dose-dependent decrease of the active equine MPO release was observed in comparison to the control tests (cells alone and cells + 1% DMSO).

### Myeloperoxidase Activity

#### Effects of Juglone on Equine MPO Activity

**Figure 4:**
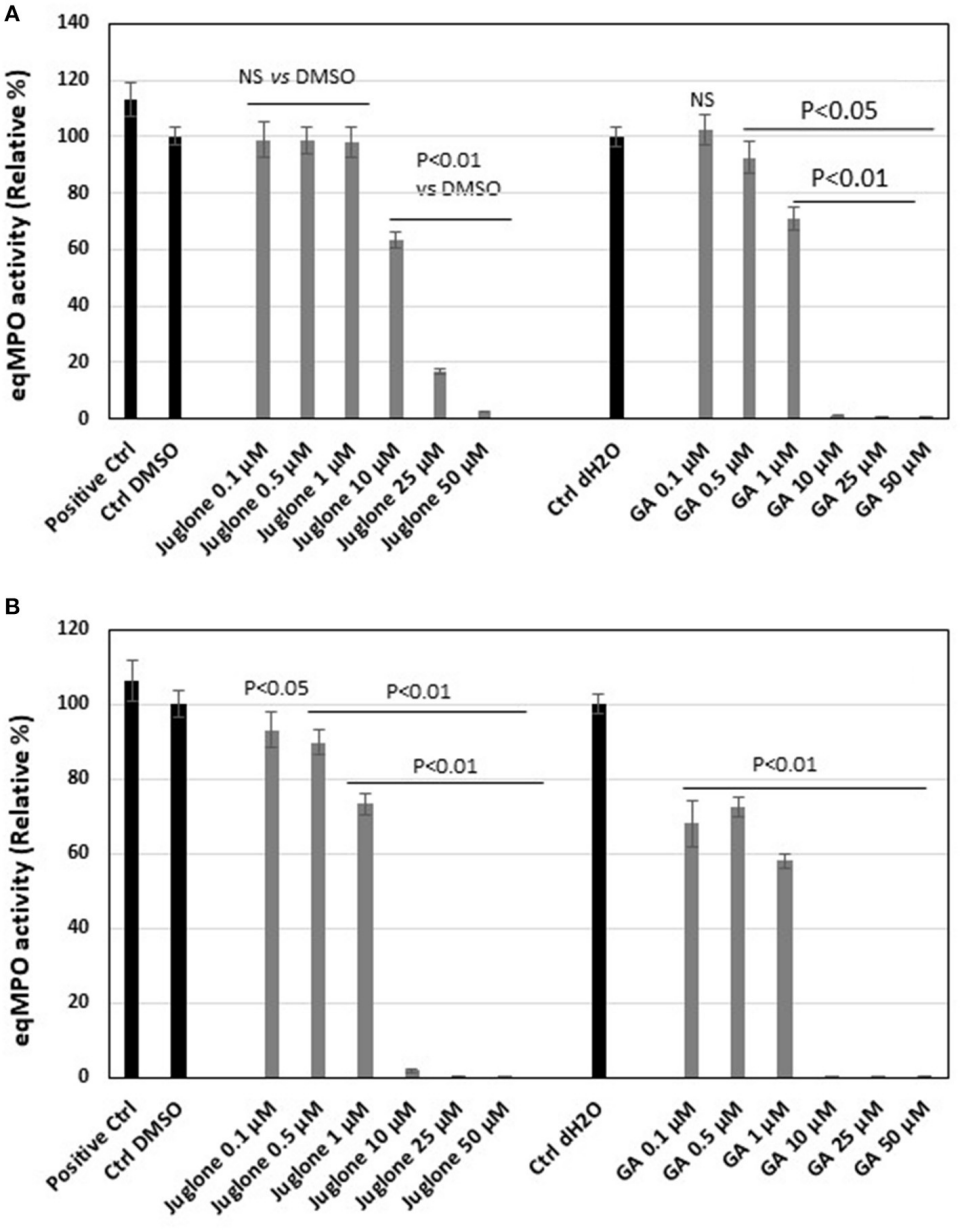
Equine myeloperoxidase (MPO) activity and effect of increasing concentrations of juglone vs. gallic acid taken as the reference inhibitor. **(A)** Activity of MPO measured by a classical enzymatic assay. **(B)** Activity of MPO measured by the specific immunological extraction followed by enzymatic detection (SIEFED) assay. The results are the mean ± SD (*n* = 4). *Positive Ctrl*, PBS with MPO alone; *Ctrl DMSO* and *Ctrl dH*_2_*O* contained MPO + 1% dimethyl sulfoxide (DMSO) or distilled H_2_O (dH_2_O), vehicles of juglone and gallic acid, respectively. Values of *p* are vs. DMSO or dH_2_O control taken as 100% activity of MPO.

The classical assay showed that, upon addition of increasing concentrations of juglone, a dose-dependent decrease of MPO activity was observed (Figure 4A). With the highest concentrations, 25 and 50 μM, the inhibition reached 85 and 95%, respectively. The inhibition values were significant (*p* < 0.01) vs. the DMSO control for the 10, 25, and 50 μM juglone concentrations. Compared to gallic acid, taken as the reference molecule, the inhibition values obtained with juglone were less pronounced. The use of the solvent of juglone (1% DMSO) and of gallic acid (1% distilled water) did not significantly influence the MPO activity compared to the MPO in PBS (positive control).

The SIEFED assay, allowing the elimination of the excess of juglone before the measurement of the MPO activity, showed a significant (*p* < 0.01 vs. the DMSO control) dose-dependent inhibition for the three highest juglone concentrations of 10, 25, and 50 μM (Figure 4B). But the inhibition was lower compared to similar concentrations of gallic acid. As for the classical assays, 1% DMSO and distilled water (dH_2_O, 1%) used to prepare gallic acid did not significantly influence the MPO activity compared to the PBS control.

#### Effects of Juglone on Human MPO Activity

**Figure 5:**
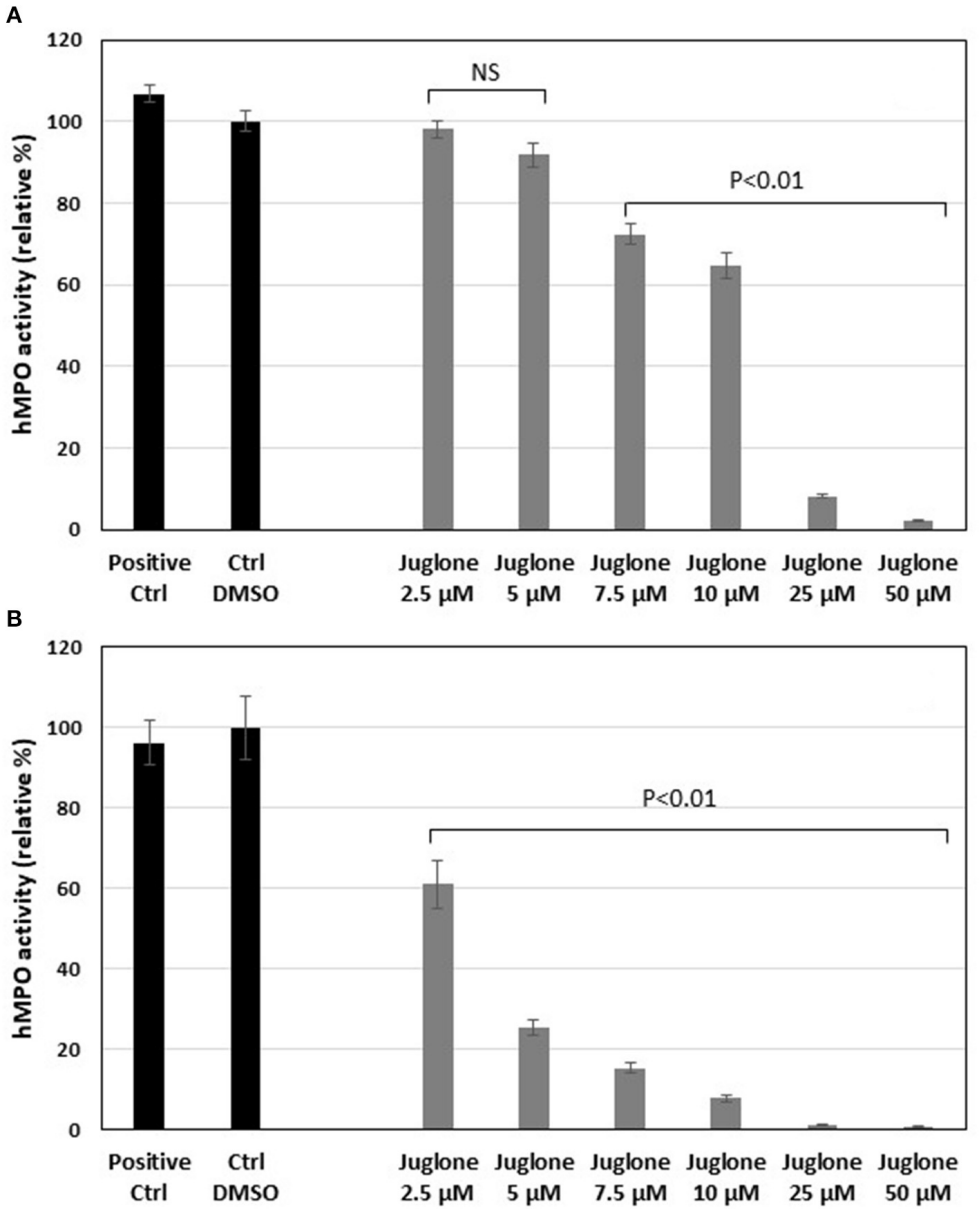
Effects of increasing concentrations of juglone on human myeloperoxidase (MPO) measured by a classical enzymatic assay **(A)** or by the specific immunological extraction followed by enzymatic detection (SIEFED) assay **(B)**. The results are the mean ± SD (*n* = 4). *Positive Ctrl*, PBS with MPO alone; *Ctrl DMSO*, control with 1% dimethyl sulfoxide (DMSO). Values of *p* are vs. DMSO control taken as 100% activity of MPO. *NS*, not significant.

As for the equine MPO, juglone exerted a significant inhibition on the human MPO activity (Figure 5). In the classical assay, the inhibition became significant (*p* < 0.01 vs. the DMSO control) starting from 7.5 μM to the highest concentrations of 25 and 50 μM (Figure 5A). In the SIEFED assay, the inhibition was significant for all the tested concentrations of juglone (*p* < 0.01 vs. the DMSO control). MPO activity was completely inhibited at 25 and 50 μM (Figure 5B).

As shown in Supplementary Figure 1, in the absence of MPO, juglone (25 or 50 μM) did not react with H_2_O_2_ (no fluorescence). Likewise, no fluorescence was detected when H_2_O_2_ was not added in the complete system (MPO, juglone, Amplex Red, and nitrite) (negative Ctrl).

### Docking of Juglone on Human MPO

**Figure 6:**
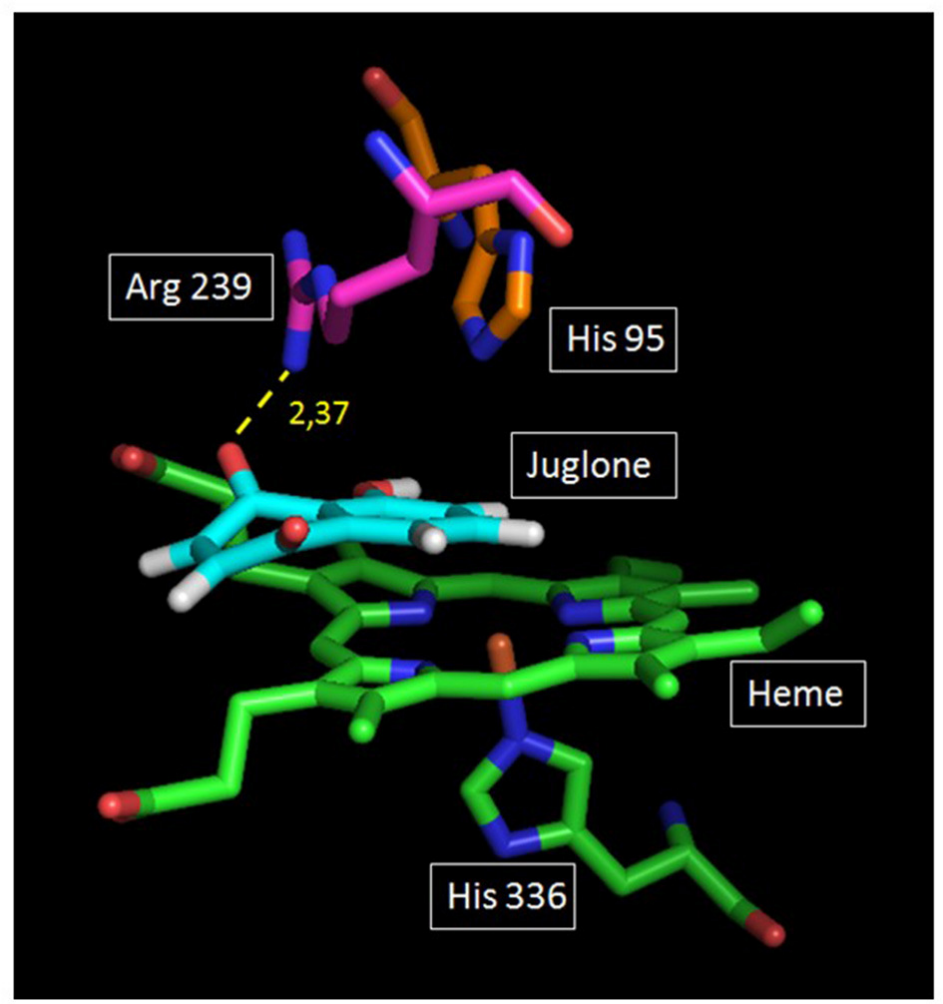
Plausible docking solution of juglone and human myeloperoxidase (MPO) analyzed by GOLD. *Green* and *blue atoms* represent the heme and the amino acid (His-336) located below the heme. *On top of the heme* are two other amino acids of the apoprotein structure (Ar-239 and His-95). Juglone is in *light blue color*. The *yellow dashed line* represents the juglone–MPO Arg-239 bond.

The docking of juglone was carried out on the well-known protein structure of human MPO (obtained by X-ray) (39). Figure 6 shows good stacking of the juglone structure on the MPO enzyme site, with a planar configuration of juglone. As shown, juglone was bound to Arg-239 by a 2.37-Å hydrogen bond between its ligand H atom and an N atom of the MPO Arg-239 residue (yellow dashed line). From this configuration, there is no interaction between the hydrogen atom of juglone and the heteroatom of His-95, another MPO residue present in the active site.

## Discussion

Laminitis is generally observed in horses when exposed to black walnut shavings. It was hypothesized that juglone, a compound found in all parts of plants of the walnut tree family (*Juglans*), was considered as an important toxic component involved in laminitis induction (40). Chiavaccini et al. (41) demonstrated that oral administration of BWE induced an early leukocyte infiltration in the colonic mucosa of horses (42). The changes in the colonic mucosa may allow the absorption of several molecules of intestinal origin, exacerbating systemic inflammation and possibly leading to distant tissue injury such as laminitis. Juglone was identified in the nuts and bark, but not in the heartwood of *Juglans nigra* mainly used for shavings (8, 12, 15). It was concluded that BWE was “laminitogenic,” but the exact implication of juglone remains unclear. However, other studies showed that juglone, when administrated *per os* to ponies, caused symptoms of laminitis inconsistently (16). Topical application of juglone to the equine digit caused local skin irritation, and intravenous administration caused acute pulmonary edema (15).

Although the role of juglone in laminitis remains controversial, a possible target of the molecule, through its naphthoquinone structure, is the modulation of the oxidant stress involved during the pathology. Indeed, during the early phase of laminitis, MPO, a pro-oxidant enzyme of neutrophils, and neutrophil elastase were present in plasma, skin, and laminar tissue, confirming the previously reported main role of neutrophils in the pathophysiology and in oxidative stress (4, 13, 14, 43).

Juglone, depending on the redox state in the cellular environment and the cell type, can behave as an antioxidant or a pro-oxidant agent (21). Naphthoquinone compounds, like juglone, can inhibit oxidant reactions by quenching ROS, inhibiting ROS-producing enzymes, and chelating transition metal ions (like Fe^2+^) by a hydrogen atom transfer mechanism (21, 44–46). On the other hand, juglone is also cytotoxic and possesses antitumor and antimicrobial properties (17, 18, 20, 21). Like other quinones, the cytotoxicity of juglone includes redox cycling and reaction with glutathione (GSH), an endogenous antioxidant. Redox cycling represents a cyclic process of the reduction of a compound followed by the oxidation of the reaction product and the simultaneous generation of ROS (21, 45). Juglone enhances lipid peroxidation through this process (47). With GSH, juglone forms adducts, causing GSH depletion, interfering with endogenous antioxidant availability (48, 49). Taking into account both the oxidant and antioxidant properties of juglone, its protective and damaging effects can be expected.

In the present work, the effect of juglone was first studied on the ROS production by neutrophils. A significant reduction of ROS release by neutrophils was measured by CL (Figure 1) and EPR (Figure 2). The CL technique was used to measure the total ROS produced by neutrophils, which were activated by PMA, a stimulating agent active on NADPH oxidase, the enzyme responsible for the production of superoxide anion, the precursor of the other ROS (33). Juglone strongly inhibited the total ROS production. EPR, combined with the DMPO spin trapping technique, is specific for superoxide anion (O${}_{2}^{-}$) detection and confirms that juglone decreased and even suppressed the production of superoxide anion radicals by the neutrophils. This inhibitory effect was not due to a cytotoxic effect of the molecule toward neutrophils since the number of dead cells was not significant between the control and juglone-treated cells, as attested by the Trypan blue exclusion test. Moreover, the use of juglone instead of PMA to trigger the ROS production by PMNs did not induce light emission, suggesting the absence of oxidant properties in this neutrophil model.

These inhibitory activities can be related to the antioxidant effects of juglone described by Ahmad and Suzuki in 2019 (21). Antioxidants reduce ROS, and the balance between ROS and antioxidants defines oxidative stress. Accumulating evidences suggest that the antioxidant properties of juglone are useful in combating oxidative stress-related diseases (like Alzheimer's in human medicine) (21, 50, 51). Zhou et al. demonstrated that juglone increased the activity of superoxide dismutase (SOD) and decreased oxidative stress in the liver of rats (51). But juglone could act in an indirect manner at the level of NADPH oxidase (Nox2), the enzyme responsible for the production of superoxide anion, the first activated species in the ROS cascade. Nox2 becomes active when its cytosolic components are phosphorylated at the level of serine residues, translocated, and then could assemble with flavoprotein b in the plasma membrane. A peptidyl prolyl *cis*/*trans* isomerase, Pin1, intervenes in the phosphorylation of the serine residues, and juglone has been reported to inhibit the pathway of Pin1, as demonstrated in CL097-induced priming of fMLP–neutrophil ROS production (52) and lipopolysaccharide (LPS)-induced priming of ROS production by neutrophils (53). It may be suggested that, also in neutrophil stimulation by PMA, juglone could interfere with the Pin 1 pathway, reducing indirectly the Nox2 activity.

On the basis of the CL and EPR spin trapping results, confirming the ROS-scavenging activity of juglone, we decided to investigate its action on the degranulation of MPO by neutrophil activated using CB/fMLP and directly on the activity of this enzyme.

The degranulation of neutrophils was obtained upon stimulation with the CB/fMLP system: CB acts on the cytoskeleton at the level of the actin filaments and is used with fMLP to potentiate neutrophil degranulation (36). In the supernatant of these activated neutrophils, the measurement of active MPO showed that juglone was effective at inhibiting the release of this enzyme, but with a variable effect from one neutrophil batch to another, as shown in Figure 3. Inhibition by juglone was also observed on purified equine MPO by acting either on the enzyme itself or on the oxidant species released during enzyme activity (Figure 4A). But the SIEFED technique (Figure 4B) demonstrates that juglone can act directly at the level of the catalytic site of MPO. This technique allows binding of the MPO present in the sample by specific immobilized antibodies, then a washing up is done before revealing the enzymatic activity. If inhibition of MPO persists after the elimination of juglone, this means that the molecule remained bonded to the captured enzyme (35). Similar inhibitory effects were also observed with human MPO (Figures 5A,B), which allowed us to use human MPO for a docking study as the enzyme structure has been elucidated by X-ray crystallography (39), which was not the case for equine MPO. This docking study showed a good stacking of juglone above the porphyrin ring of MPO and a hydrogen bond with Arg-239 (Figure 6). No interaction was found between a hydrogen atom of juglone and the heteroatom of His-95 on the apoprotein present in the active site. Nevertheless, the planar configuration of juglone and the link established with the Arg-239 residue confer a strong stability in the active site, hindering access to the iron-bearing catalytic site for H_2_O_2_, the normal MPO substrate, and inhibiting the important step of the formation of a π-cation radical state on the porphyrin ring (54). In this way, the formation of the highly oxidant HOCl molecule is impaired.

By generating potent oxidant molecules, MPO is a dangerous enzyme responsible for damage in acute and chronic inflammation pathologies, and its inhibition could be beneficial. Several natural compounds such as curcumin and resveratrol are candidates for MPO inhibition (55, 56). Our study demonstrates that juglone, another natural compound, might be a useful candidate in inflammation pathologies.

## Conclusion

The present work using an *in vitro* model of neutrophil degranulation and MPO activity indicates, for the first time, that juglone has anti-inflammatory effects, rather than pro-inflammatory ones, on equine neutrophils and MPO. Through its antioxidant properties, by scavenging the ROS produced by PMA-stimulated PMNs and by inhibiting the MPO activity, inhibitory effects confirmed by the docking study, juglone would be protective in the BWE model of equine laminitis rather than damaging, at least if we focus on the modulation of neutrophil activation. But concerning the oxidant neutrophil activity, another mode of action should be considered for juglone: a pathway involving NADPH oxidase. Finally, it should be taken into account that juglone, in *in vivo* conditions, could contribute with other compounds of BWE to intestinal inflammation and to the resorption of pathogen-associated molecular patterns (PAMPs) able to induce the systemic and local inflammation that characterizes the BWE model of equine laminitis.

## Data Availability Statement

The original contributions presented in the study are included in the article/Supplementary Material, further inquiries can be directed to the corresponding author/s.

## Author Contributions

DS, AM-M, and NS: conceptualization. NS, AM-M, TF, and JC: methodology. AM-M, NS, and TF: investigation. AM-M, DS, GD-D, and NS: writing-original draft preparation. AM-M, DS, JC, TF, and GdR: writing-review and editing. DS and AM-M: supervision. All authors contributed to the article and approved the submitted version.

## Conflict of Interest

The authors declare that the research was conducted in the absence of any commercial or financial relationships that could be construed as a potential conflict of interest.

## Supplementary Material

Supplementary Figure 1 Effect of hydrogen peroxide (H_2_O_2_) on the reactivity of increasing concentrations (from 0.1 μM to 50 μM) of juglone in the complete reaction mixture (nitrite and Amplex Red) but without MPO (H_2_O_2_, no MPO). Positive Ctrl MPO and DMSO: complete system).
