# Supplementary figures and images for: Effects of Juglone on Neutrophil Degranulation and Myeloperoxidase Activity Related to Equine Laminitis
Source: Front Vet Sci. 2021 Jul 16;8:677675. doi: 10.3389/fvets.2021.677675 (PMC8322847; doi:10.3389/fvets.2021.677675)

# Effect of H2O2 on the reactivity of Juglone

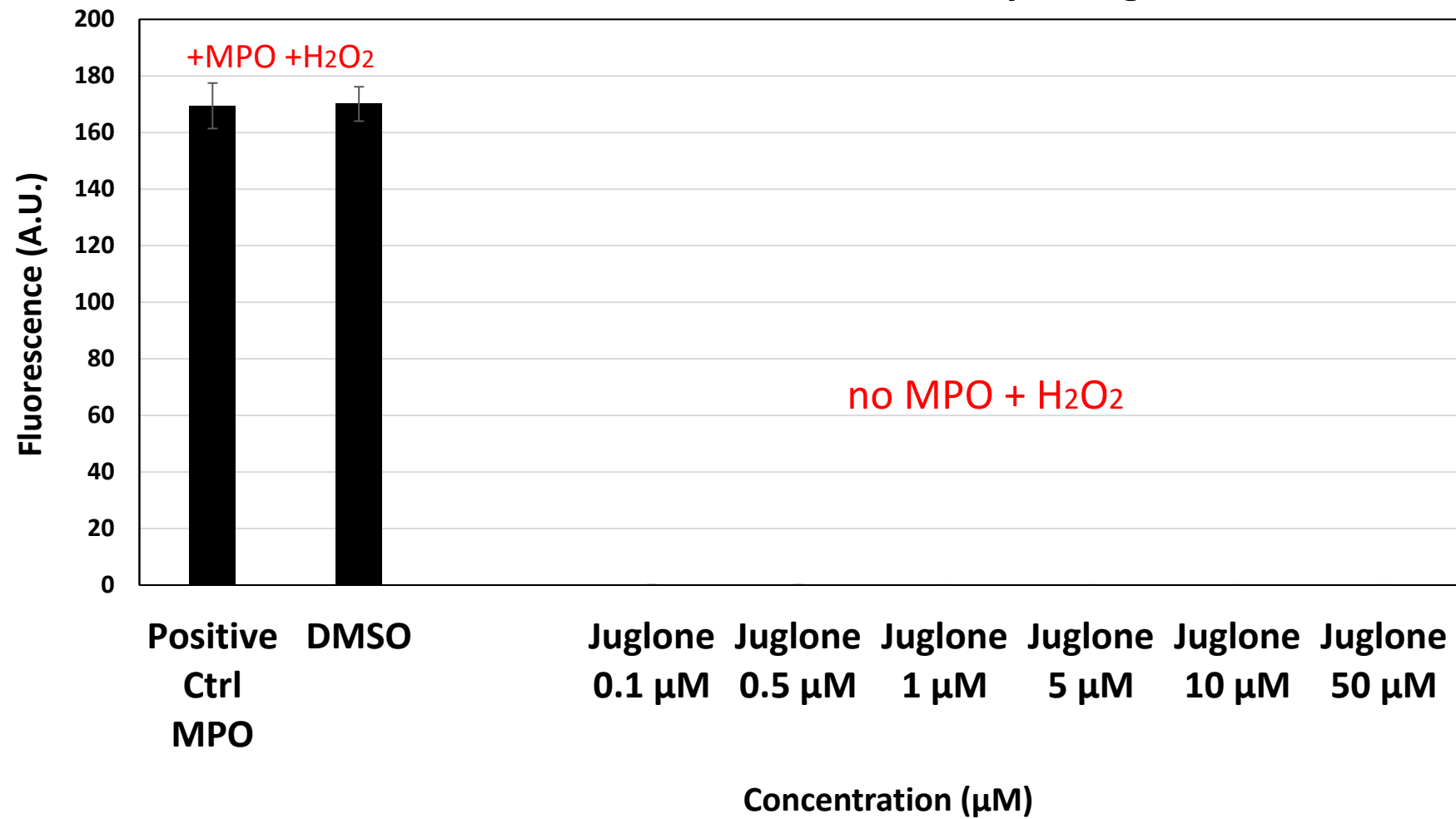

Supplement: Supplementary Figure 1 — Effect of hydrogen peroxide (H2O2) on the reactivity of increasing concentrations (from 0.1 μM to 50 μM) of juglone in the complete reaction mixture (nitrite and Amplex Red) but without MPO (H2O2, no MPO). Positive Ctrl MPO and DMSO: complete system). [file Image_1.pdf]
